# Experience using mTOR inhibitors for subependymal giant cell astrocytoma in tuberous sclerosis complex at a single facility

**DOI:** 10.1186/s12883-021-02160-5

**Published:** 2021-03-31

**Authors:** Kyoichi Tomoto, Ayataka Fujimoto, Chikanori Inenaga, Tohru Okanishi, Shin Imai, Masaaki Ogai, Akiko Fukunaga, Hidenori Nakamura, Keishiro Sato, Akira Obana, Takayuki Masui, Yoshifumi Arai, Hideo Enoki

**Affiliations:** 1 Department of Neurosurgery, Seirei Hamamatsu General Hospital, 2-12-12 Sumiyoshi, Nakaku, Hamamatsu 430-8558 Shizuoka, Japan; 2 Tuberous Sclerosis Complex Board, Seirei Hamamatsu General Hospital, 2-12-12 Sumiyoshi, Nakaku, Hamamatsu 430-8558 Shizuoka, Japan

**Keywords:** Subependymal giant cell astrocytoma (SEGA), Tuberous sclerosis complex (TSC), Mammalian target of rapamycin inhibitor (mTORi), Surgery, Hydrocephalus, Interdisciplinary team

## Abstract

**Background:**

Subependymal giant cell astrocytoma (SEGA) is occasionally seen in tuberous sclerosis complex (TSC). Two main options are currently available for treating SEGA: surgical resection or pharmacotherapy using mammalian target of rapamycin inhibitors (mTORi). We hypothesized that opportunities for surgical resection of SEGA would have reduced with the advent of mTORi.

**Methods:**

We retrospectively reviewed the charts of patients treated between August 1979 and July 2020, divided into a pre-mTORi era group (Pre-group) of patients treated before November 2012, and a post-mTORi era group (Post-group) comprising patients treated from November 2012, when mTORi became available in Japan for SEGA. We compared groups in terms of treatment with surgery or mTORi. We also reviewed SEGA size, rate of acute hydrocephalus, recurrence of SEGA, malignant transformation and adverse effects of mTORi.

**Results:**

In total, 120 patients with TSC visited our facility, including 24 patients with SEGA. Surgical resection was significantly more frequent in the Pre-group (6 of 7 patients, 86 %) than in the Post-group (2 of 17 patients, 12 %; *p* = 0.001). Acute hydrocephalus was seen in 1 patient (4 %), and no patients showed malignant transformation of SEGA. The group treated using mTORi showed significantly smaller SEGA compared with the group treated under a wait-and-see policy (*p* = 0.012). Adverse effects of pharmacotherapy were identified in seven (64 %; 6 oral ulcers, 1 irregular menstruation) of the 11 patients receiving mTORi.

**Conclusions:**

The Post-group underwent surgery significantly less often than the Pre-group. Since the treatment option to use mTORi in the treatment of SEGA in TSC became available, opportunities for surgical resection have decreased in our facility.

## Background

Subependymal giant cell astrocytoma (SEGA) is often seen in patients with tuberous sclerosis complex (TSC), with a reported frequency of 1–27.4 % [1–7]. Management of SEGA used to involve resection in open cranial surgery [8, 9], because SEGA may cause hydrocephalus [10], and, in rare cases, malignant transformation [11] or intra-tumoral hemorrhage [12]. Patients with SEGA may even undergo laser ablation [13, 14] or endoscopic surgery [15–19]. However, as TSC is a systemic disease, most patients with TSC suffer from not only SEGA, but also pathologies such as renal, pulmonary, dermatological, and ophthalmological diseases. This is because the mechanisms underlying TSC involve abnormal upregulation of the mammalian target of rapamycin (mTOR) pathway in patients with TSC and subsequent tumor growth in various organs. Interdisciplinary medical management is thus required for the treatment of TSC [3, 20, 21]. The importance of interdisciplinary medical management for TSC is nowadays acknowledged by many physicians who treat patients with TSC. This is probably due to the availability of mTOR inhibitors (mTORi) [7]. As mTORi show efficacy against not only SEGA, but also pathologies such as renal angiomyolipoma [22, 23], epileptic seizures [24], neuropsychiatric disorders [25], and lymphangioleiomyoma [26, 27] over a wide age range [28], the situation surrounding patients with TSC has become more complicated. Patients with SEGA should thus be followed-up by a well-organized interdisciplinary team including internal and surgical physicians [3, 20]. The current treatment strategy for SEGA entails two treatment options: surgical removal or a pharmacotherapeutic approach using mTORi [29]. Theoretically, given these two options, the probability of a patient undergoing resective surgery would be reduced. In this study, we hypothesized that opportunities for surgical treatment of SEGA would have decreased since the introduction of mTORi. The purpose of this study was thus to compare the frequency of surgical treatment for SEGA between the pre- and post-mTORi eras as a primary outcome measure. We also monitored the size of SEGA and the adverse effects of mTORi, and examined differences in treatment goals for SEGA before and after the advent of mTORi by reviewing rates of acute hydrocephalus, malignant transformation and reasons for surgery as secondary outcome measures.

## Methods

### Study design

Participants in this cross-sectional, observational, non-randomized study were identified from a retrospective review of medical charts for patients treated between August 1979 and July 2020 by the Tuberous Sclerosis Board at Seirei Hamamatsu General Hospital. This study was conducted as an analytical comparative study, and not as a descriptive study, so it did not represent a case-series study. Moreover, since results were obtained retrospectively and interventions were not applied to patients to obtain the data for this study, it did not represent an interventional study.

### Clinical information

We retrospectively reviewed patients with TSC who were diagnosed genetically or clinically in accordance with the clinical diagnostic criteria [30]. Inclusion criteria were: (1) uni- or bilateral SEGA observed on magnetic resonance imaging (MRI) of the brain; (2) MRI or computed tomography (CT) of the brain performed every 1–3 years; and (3) follow-up at > 1 year. The neuroradiological criterion was included in accordance with the recommendations of the International Tuberous Sclerosis Complex Consensus Conference [31, 32]. SEGAs in this study were defined as lesions within the cerebral ventricles with maximum diameter ≥10 mm. We regarded lesions with maximum diameter < 10 mm as subependymal nodules and excluded those cases from the present study [33, 34].

### Outcome measurements

Among the enrolled patients, those patients diagnosed with SEGA before November 2012 were categorized into the pre-mTORi era group (Pre-group). Patients diagnosed with SEGA after November 2012 were categorized into the post-mTORi era group (Post-group). The cut-off of November 2012 was applied as the time when mTORi (everolimus) became available in Japan. Patients in the Post-group who exhibited tumor growth on serial MRI or CT took everolimus at 3 mg/m^2^/day. If no tumor growth was observed and other pathologies did not require everolimus, we adopted a wait-and-see policy. When a patient exhibited adverse effects from everolimus, the dose was reduced to every other day to 3 days/week, depending on symptoms. If adverse effects lasted a long time, patients could suspend the use of everolimus for a period of up to 1 month. Patients who stopped use of everolimus for > 1 month were excluded from analysis in this study. We compared groups in terms of surgical and mTORi treatment.

We also reviewed the rate of acute hydrocephalus, the reason for surgical resection, rates of SEGA recurrence in the surgical and non-surgical subgroups, and the rate of malignant transformation. We monitored SEGA size before and after mTORi administration by serial MRI or CT for patients who receiving mTORi. Serial neuroimages were obtained every 1–3 years, in line with the recommendations of the International TSC consensus conference [32]. SEGA size was defined as the largest diameter on axial, coronal or sagittal MRI or axial CT. We also reviewed adverse effects attributed to mTORi. For the reference, we also measured SEGA size just before SEGA resective surgery in the Pre-group.

### Statistical analysis

For statistical analyses of clinical data, we used Student’s *t*-tests, and the Mann-Whitney U-tests, as well as Fisher’s exact probability tests to compare patients who underwent resective surgery in the pre- and post-mTORi eras. Values of *p* < 0.05 were considered statistically significant. All statistical analyses were performed using Sigma Plot version 14.0 software (Systat Software, San Jose, CA, USA).

## Results

### Clinical information

A total of 120 patients with TSC visited our facility, including 23 patients (19 %) with SEGA who met the inclusion criteria for the study. One patient (Patient 4) had bilateral SEGAs. In that patient, one SEGA was surgically removed in the pre-mTORi era and the other was treated using mTORi. This patient was therefore counted twice. As a result, we analyzed 24 patients (16 males, 8 females) in total (Table 1). Age at presentation to our hospital (*p* = 0.031) and current age (*p* = 0.002) were both significantly lower in the Post-group than in the Pre-group.

**Table 1:** Clinical information for the pre- and post-mTORi eras

	pre-mTORi era	post-mTORi era	*p*-value
Age at SEGA removal operation (years)	6, 6-19 years, mean 10, median 8.5, SD 5.02	2, 1-9 years, mean 5, median 5, SD 5.65	0.407
Age at mTORi administration (years)	n/a	11, 0.8-24 years, mean 12.3, median 14, SD 8.17	n/a
Age at presentation to our hospital	7, 9-41 years, mean 22.6, median 19, SD 11.3	17, 0.8-23 years, mean 10.5, median 12, SD 7.7	0.031*
Current age (years)	7, 19-43 years, mean 29.9, median 29, SD 8.78	17, 1-29 years, mean 14, median 15, SD 8.16	0.002*
Sex (male / female)	3 f / 4 m	5 f / 12 m	

*mTORi* Mammalian target of rapamycin inhibitor, *n/a* Not available, *SD *Standard deviation, *SEGA *Subependymal giant cell astrocytoma

### Outcome measurements

Six of the seven patients in the Pre-group underwent surgical resection of SEGA, while a wait-and-see policy was adopted for the remaining patient. In the Post-group, two of the 17 patients underwent surgical resection. An mTORi was administered (11 patients) or a wait-and-see policy was adopted (four patients) in the remaining 15 patients. Surgical resection of the SEGA was thus significantly more frequent in the pre-Group (*p* = 0.001).

Clinical characteristics of the eight patients who underwent surgical resection across both eras are shown in Table 2. Five of these eight patients displayed an enlarged lateral ventricle on neuroimaging, but only one patient (Patient 3) exhibited symptoms of increased intracranial pressure (iICP), was diagnosed with symptomatic acute hydrocephalus and underwent emergency surgery. All patients who did not undergo surgery were asymptomatic. Thus, only one of the 24 patients (4 %) presented with acute iICP.

**Table 2:** Clinical characteristics of 8 cases with surgically resected SEGA associated with TSC

Patient no.	Sex	Age at operation	Date of operation	Approaches to the SEGA	Symptoms of SEGA (reason for surgery)
1	M	6	pre-mTORi era	surgical removal	enlarged lateral ventricle (prophylactic)
2	M	10	pre-mTORi era	surgical removal (+ frontal lobe focus resection)	enlarged lateral ventricle (prophylactic)
3	F	19	pre-mTORi era	surgical removal (emergency)	acute hydrocephalus (cure of iICP)
4	F	6	pre-mTORi era	VP shunt, then surgical removal	enlarged lateral ventricle (prophylactic)
5	M	12	pre-mTORi era	surgical removal	none (prophylactic)
6	M	7	pre-mTORi era	surgical removal, then VP shunt	enlarged lateral ventricle (prophylactic)
7	M	9	post-mTORi era	surgical removal	none (prophylactic)
8	F	1	post-mTORi era	surgical removal (+ total corpus callosotomy)	none (prophylactic)

*F* Female, *M* Male, *SEGA* Subependymal giant cell astrocytoma, *VP shunt* Ventriculoperitoneal shunt, *iICP* Increased intracranial pressure

Only one patient (Patient 4) experienced SEGA growth, arising on the contra-surgical side. We administered mTORi for this tumor growth, and the tumor subsequently decreased in size. No cases of recurrence or malignant transformation were seen in any other patients.

**Fig. 1:**
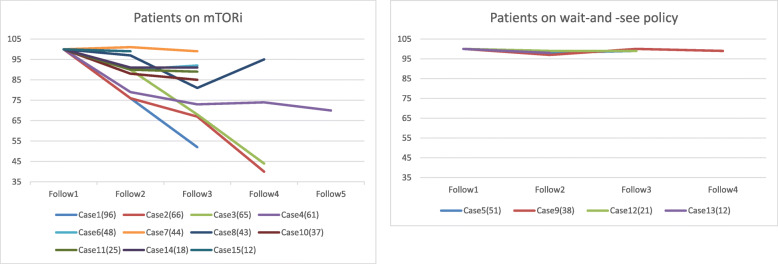
At the time of latest follow-up, patients receiving mammalian target of rapamycin inhibitors (mTORi) showed reduced size of the SEGA compared with the wait-and-see policy group (*p* = 0.012). The number in parentheses after each case shows total follow-up in months. Neuroradiological follow-up was performed every 1–3 years

Size of the SEGA treated by mTORi and size of the SEGA treated under a wait-and-see policy are shown in Fig. 1. SEGA size in the group administered mTORi ranged from 10.19 mm to 23.18 mm (mean, 12.87 mm; SD, 3.68 mm; median, 11.42 mm). SEGA size in the wait-and-see policy group ranged from 10.23 mm to 15.42 mm (mean, 12.72 mm; SD, 2.69 mm; median, 12.6 mm). The group treated by mTORi showed a significantly reduced SEGA size compared with the group treated using a wait-and-see policy (*p* = 0.012). Adverse effects from mTORi were identified in seven (64 %) of the 11 patients. Of these, six patients (54 %) exhibited oral ulcers and one (10 %) exhibited irregular menstruation. SEGA size just before resective surgery ranged from 10.98 mm to 31.1 mm (mean, 19.39 mm; SD, 7.65 mm; median, 18.62 mm). SEGA size in the group treated by resective surgery was not significantly larger than that in the mTORi treatment group (*p* = 0.054). These data were only for the reference because neuroimages could not be collected just before the resective surgery from two of the patients and thus were not included in this measurement.

## Discussion

Obviously, the Post-group used mTORi more frequently than the Pre-group in our facility. This study, however, confirmed that the frequency of surgical resection was indeed reduced following the advent of mTORi.

In this study, only one of the 24 patients (4 %) clinically exhibited acute iICP symptoms. However, other studies have reported a high rate of acute hydrocephalus, even with high mortality rates [9]. In the present study, only one patient showed acute hydrocephalus and no mortality occurred. SEGA size is related to the rate of hydrocephalus [35] and early diagnosis and early treatment by the mTORi are reported to successfully prevent hydrocephalus [36]. As age at presentation to our hospital was lower in the Post-group than in the Pre-group in this study, SEGA identification while still small might explain the lower rate of acute hydrocephalus.

Many reports have stated that SEGA grows near and occludes the foramen of Monro, leading to obstructive hydrocephalus. However, we know that some cases show apparent occlusion of the foramen on MRI while not exhibiting iICP symptoms and others do not show clear occlusion of the foramen but still exhibit symptoms of iICP. The theory of obstructive and communicating hydrocephalus is now known to be inadequate for explaining the pathophysiology of hydrocephalus. More complicated causative mechanisms must therefore be involved in hydrocephalus, such as brain compliance and osmotic pressure due to cerebrospinal fluid protein [37–40]. However, the classical theory is still often applied to the mechanisms underlying hydrocephalus with SEGA. The actual rate of acute hydrocephalus remains unclear, with a wide variation in reported values of 38–80 % [8, 9, 29, 41–44]. A huge discrepancy exists between the present study (4 %) and those previous investigations. This was probably due to differences between treatment by an interdisciplinary team or a single department. A single department such as neurosurgery may see a patient with SEGA who visits their department with iICP symptoms. However, as tumor growth is age-dependent and the natural history of this pathological entity is well-known [45], expertise ranging from pediatrics to adult neurology and neurosurgery is required in terms of dealing with the hydrocephalus. Surgical and internal medical treatments have both positive and negative aspects [36, 46–48]. Among these, we must choose the best treatment for patients with SEGA in a well-organized interdisciplinary team. The present study found that we mostly treated SEGA prophylactically for fear of possible hydrocephalus, with only one patient with symptomatic hydrocephalus undergoing emergency tumor resection. Considering the high mortality and morbidity rates in patients with SEGA [45, 49], shifting the treatment concept of SEGA from emergent hydrocephalus treatment by a single department to prophylactic treatment by an interdisciplinary team appears warranted.

Some differences between SEGA with and without iICP, such as variations in immunohistochemical features, genetic differences, and anaplastic features [50–52] or protein-producing functions [53, 54], may relate to brain compliance or osmotic pressure. However, this study could not address this question. In addition, whether mTORi ceased or reduced the protein-producing function of SEGA was also unclear in this study. As a perspective on future investigations, studies involving multiple facilities are required to clarify risk factors for SEGA with iICP in advance of determining optimal treatments.

## Conclusions

Since the treatment option to use mTORi in the treatment of SEGA in TSC became available, opportunities for surgical resection have decreased in our facility.

## Data Availability

All data in support of our findings are presented within this manuscript.

## Abbreviations

CT: Computed tomography
iICP: Increased intracranial pressure
mTOR: Mammalian target of rapamycin
mTORi: Mammalian target of rapamycin inhibitor
MRI: Magnetic resonance imaging
SEGA: Subependymal giant cell astrocytoma
TSC: Tuberous sclerosis complex
